# Fossil wood cells recorded 300 million years of Europe’s tectonic history

**DOI:** 10.1038/s41598-026-60054-3

**Published:** 2026-07-14

**Authors:** Steffen Trümper, Matthias Franz, Graciela Sosa, Alfons van den Kerkhof, Armin Zeh, Michael Tatzel, Andreas Kronz, Kirsten Techmer, Tommaso Di Rocco, Andreas Pack, Ronny Rößler

**Affiliations:** 1 Institute of Geology and Paleontology, University of Münster, 48149 Münster, Germany; 2 Geoscience Center, Georg August University, 37077 Göttingen, Germany; 3 Institut für Angewandte Geowissenschaften, Mineralogie und Petrologie, KIT – Karlsruher Institut für Technologie, 76131 Karlsruhe, Germany; 4 Museum für Naturkunde Chemnitz, 09111 Chemnitz, Germany; 5 Geological Institute, TU Bergakademie Freiberg, 09599 Freiberg, Germany

**Keywords:** Environmental sciences, Solid Earth sciences

## Abstract

Woody debris undergoes the same processes of deposition and diagenesis as its embedding sediments, making its fossilized products potentially good archives of basin evolution. We review this hypothesis by reconstructing the timelines, source and physicochemical conditions of mineralization in silicified Pennsylvanian wood from the Kyffhäuser, central Germany. Five stages of silicification are evidenced from quartz cathodoluminescence, fluid inclusions, triple-O and Si isotopes, Raman thermometry, electron-microprobe and scanning-electron analyses, and in situ U–Pb radiogenic isotope dating. (1) 304–299 Ma: permineralization of fluvial woody debris at near-surface conditions sourced from pedogenically altered pyroclasts; (2) 299–290 Ma: opal-quartz transformation during initial burial; (3) 257–260 Ma: quartz-hematite mineralization at 1 km depth related to thermal crustal relaxation; (4) c. 180 Ma: quartz formation from basinal brines upon maximum burial at 170–240°C and 3.5–5 km; and (5) 100 Ma: regional quartz-baryte mineralization following basin inversion before final exhumation in the Quaternary. The results expand the hitherto known timescales of wood mineralization, and reveal that fossil wood can record a basin’s burial history and disclose the tectonic events on the hundred-million-year timescale.

**Supplementary Information:**

The online version contains supplementary material available at 10.1038/s41598-026-60054-3.

## Introduction

Detailed knowledge of the evolution of sedimentary basins is essential for reconstructing the geological history of a region and assessing the significance of sedimentary rocks for scientific and economic purposes. However, this information can only be obtained by clarifying the drivers, courses, and timelines of deposition, diagenesis and tectonic evolution using various methods^1, 2^. In doing so, we often have to resort to different archives, such as the architecture, grain morphology, composition and geochemistry of the sediment, biostratigraphically relevant fossils, the thermal maturation, structure and chemistry of detrital and authigenic minerals, and deformation structures. The search for and use of geological archives and approaches that elucidate various aspects of basin evolution are therefore of utmost interest.

Fossil wood is a potential archive for basin analysis that has received surprisingly little attention to date, despite several advantages. First, wood became an increasingly abundant detrital component in sediments since its widespread emergence in the Devonian, due to its physicochemical resistance and occurrence in many plant groups and environments^3, 4, 5, 6^. Second, among all organic hard tissues, wood has the strongest chemical affinity to mineralize, reflected by fossil woods from various Phanerozoic basins worldwide and more than 80 mineral phases identified therein^7, 8^. Replacement and/or impregnation by silica (i.e. silicification) is predominant, mirroring silica as the most abundant oxide in the Earth’s crust and its ability to bond to the wood chemical components^9, 10, 11, 12^. Fossil wood hence occurs in various depositional facies and is subject to sediment diagenesis and deformation, while its mineralizing phases record the physicochemical conditions over various time and depth scales^13^.

In this paper, we present the value of fossil wood as an archive for reconstructing basin evolution, in particular to quantify the physicochemical conditions and age of wood burial, tissue mineralization, as well as tectonic and hydrothermal events. Various novel and classical approaches are combined here to address the topic, including cathodoluminescence (CL) microscopy, in situ U–Pb radiogenic isotope dating, fluid-inclusion analyses, oxygen- and silicon-isotope analyses of fossil-wood quartz. The study subject is silicified wood from the Kyffhäuser, a local, mountainous basement high in central Germany. These fossils, known at least since the eighteenth century, comprise trunks up to 20 m long embedded in Upper Pennsylvanian fluvial red beds^14^. Their deposition resulted from flood burial of large woody debris that derived from gymnospermous tropical dryland forests in equatorial Pangea^15^. The Kyffhäuser is the type section of a large-woody-debris-bearing facies that repeatedly formed in various basins across Europe from the late Middle Pennsylvanian–early Permian^16^, forming one of the most voluminous occurrences of silicified wood in the Northern Hemisphere. From a basin-analytical point of view, evidence of basin evolution from the Kyffhäuser fossil wood is crucial, since the Pennsylvanian host rocks belong to the initial deposits of the Central European Basin (CEB) – an economically important, intracontinental arrangement of sedimentary basins with a complex evolution from the late Carboniferous until today^17, 18^. This basin system has been extensively studied through abundant stratigraphic, sedimentological, petrological, and geophysical studies of outcrops, borehole data and core material, providing a robust database to test the significance of fossil wood in basin research.

## Results

### The Kyffhäuser red beds

**Fig. 1:**
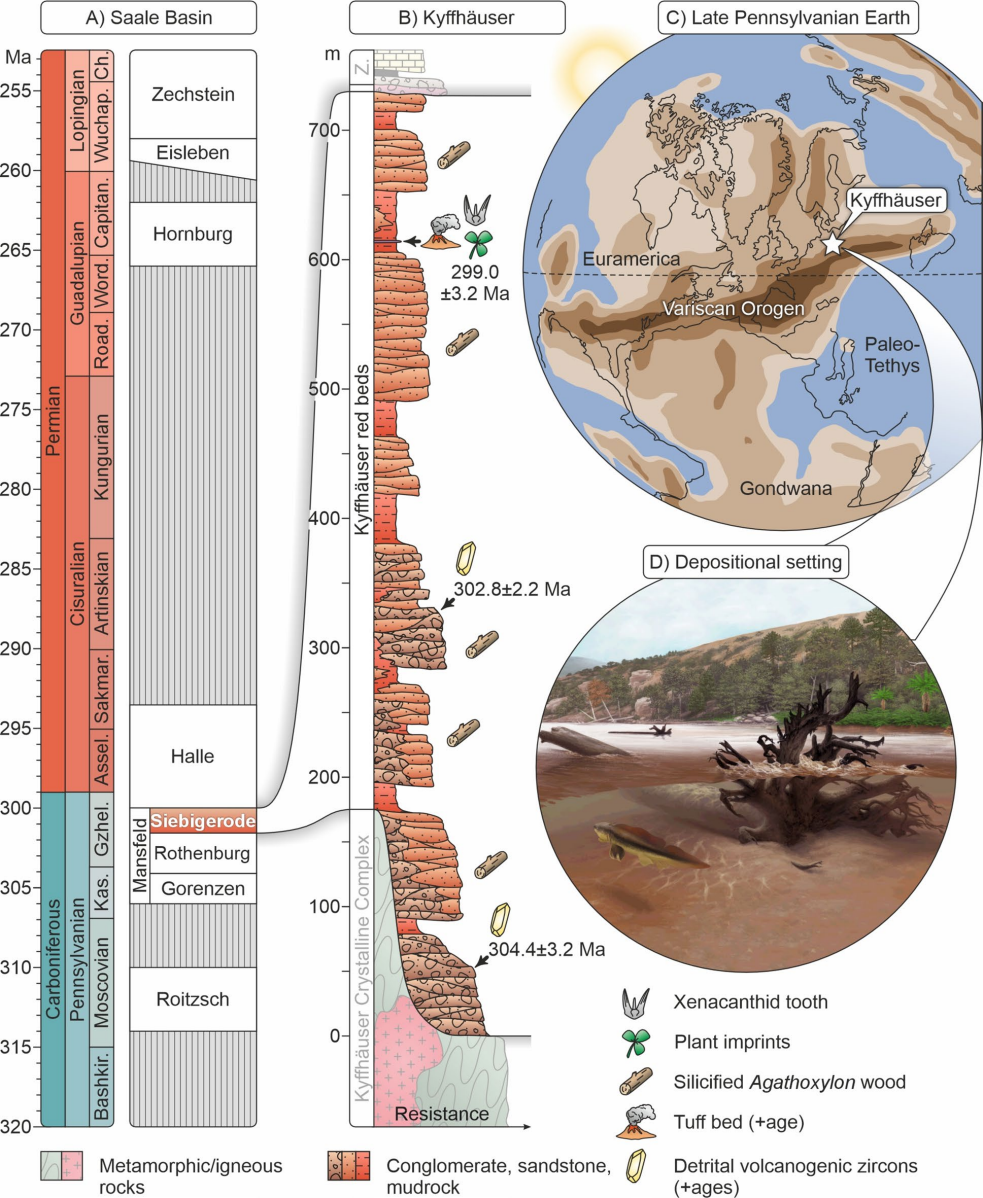
Stratigraphy and paleoenvironment of the Kyffhäuser fossil trunks. (**A**), Lithostratigraphy of the Saale Basin. (**B**), Profile of the Siebigerode Formation in the Kyffhäuser. (**C**), Paleotropical position of the study area in equatorial Euramerica. (**D**), Formation of the red beds in a fluvial dryland setting (environment reconstruction: Frederik Spindler).

**Fig. 2:**
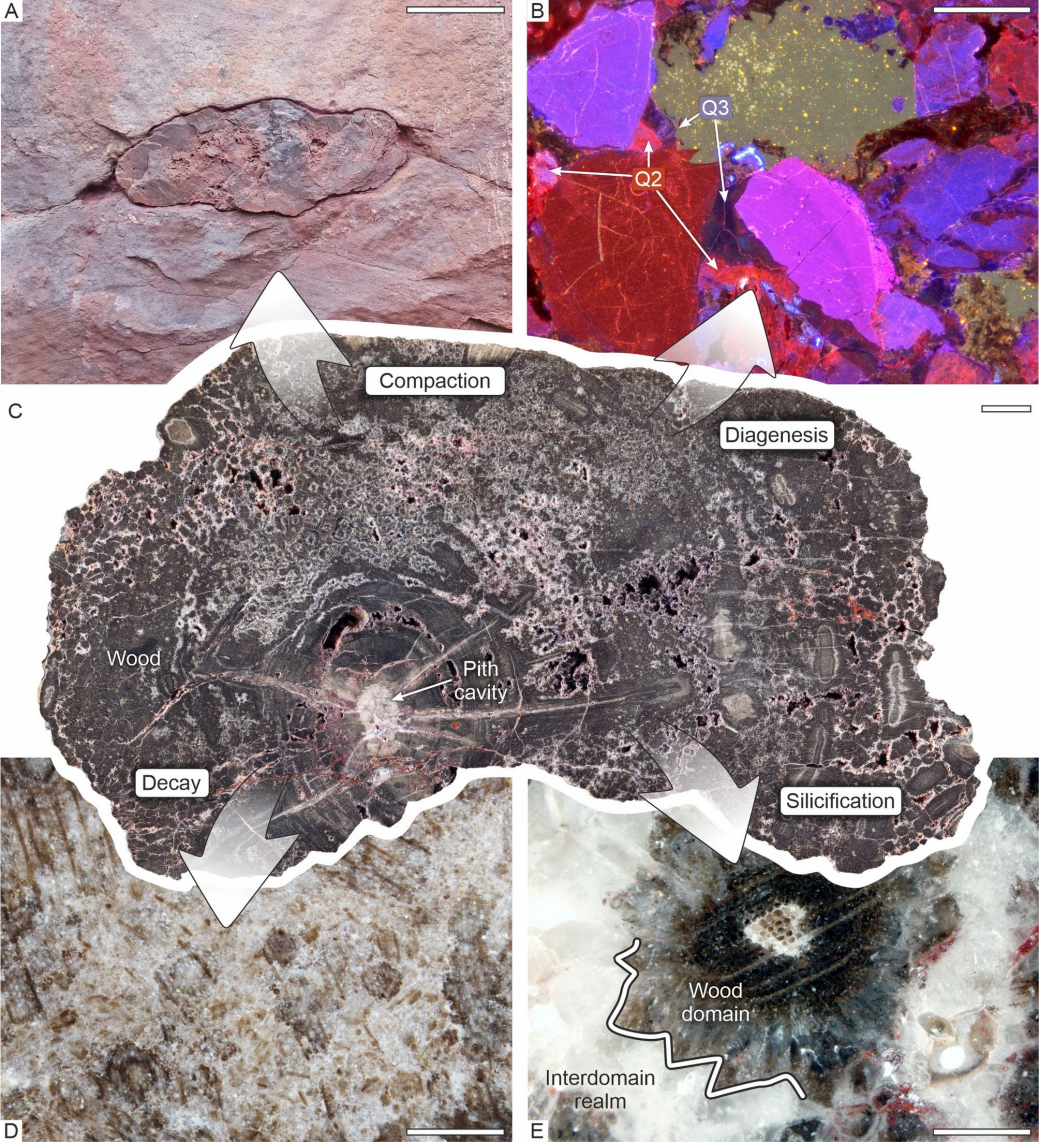
Preservation of the Kyffhäuser fossil trunks. (**A**) Petrified trunk in sandstone. (**B**) Silica cements of embedding sandstone correlate with silicification stages Q2 and Q3 of the fossil wood. (**C**) Fossil trunk in transverse section; K6194. (**D**) Occurrence of wood domains (arrows) within decomposed wood; K134a. (**E**) Detail of the petrifaction domains in transverse section; both K2097. Scales: 20 cm (**A**); 200 µm (**B**); 1 cm (**C**); 500 µm (**D**); 500 µm (**E**).

Permineralized wood of the Kyffhäuser occurs as decorticated logs, rootwads and branches that are embedded isolated and in horizontal position in > 700 m thick red beds (Fig. 1). The quartz-rich strata are assigned to the Siebigerode Formation and comprise conglomerate, sandstone and shale facies formed in a proximal fluvial channel-floodplain system of the Saale Basin^15^ (Figs. 1, 2A). This basin measured at least 120 km in length and 60 km in width, and existed along the northern margin of the strongly truncated Variscan Orogen of equatorial Pangea (Fig. 1C). Alike in other intramontane basins of central Europe, deposition of the Kyffhäuser red beds was accompanied by post-collisional volcanism^19, 20, 21^. Synsedimentary tephra fallout in the Saale Basin catchment is indicated by a crystal-rich tuff bed^15^, the presence of euhedral zircon grains, detrital kaolinite-group aggregates and argillized volcanic grains in the Kyffhäuser red beds. Multiple laser ablation inductively coupled plasma mass spectrometry (LA-ICP-MS) spot analyses of single zircon crystals from the basal and middle Kyffhäuser section yielded weighted average ^238^U/^206^Pb ages of 304.4 ± 3.2 Ma and 302.8 ± 2.2 Ma, respectively (Fig. 1B; Supplementary Data 1). These maximum depositional ages are consistent with the 299.0 ± 3.2 Ma age of the tuff bed from the upper Kyffhäuser section^15^ (Fig. 1B) and the resulting range of c. 304–299 Ma largely agrees with biostratigraphy-based correlations^22^.

The presence of chemically altered detrital grains, pervasive diagenetic cementation and secondary intragranular porosity attest to intense early- to late-stage chemical diagenesis^15^, (Fig. 2B). Early-stage pedogenesis resulted in the alteration of labile volcanic grains, i.e., kaolinization of feldspar and volcanic glass, and accumulation of clays and Fe-oxides/hydroxides in illuviation horizons. Successive burial–uplift cycles contributed to a complex diagenetic history of the Kyffhäuser red beds with a latest Jurassic maximum burial depth of c. 5,000 m^23^, followed by late Cenozoic exhumation. The burial diagenesis was punctuated by crust-scale fluid flow resulting in typical vein mineralizations in the Kyffhäuser and nearby Harz basements^24^ and identical paragenetic sequences in the Kyffhäuser red beds and wood.

### Preservation of the Kyffhäuser wood

**Fig. 3:**
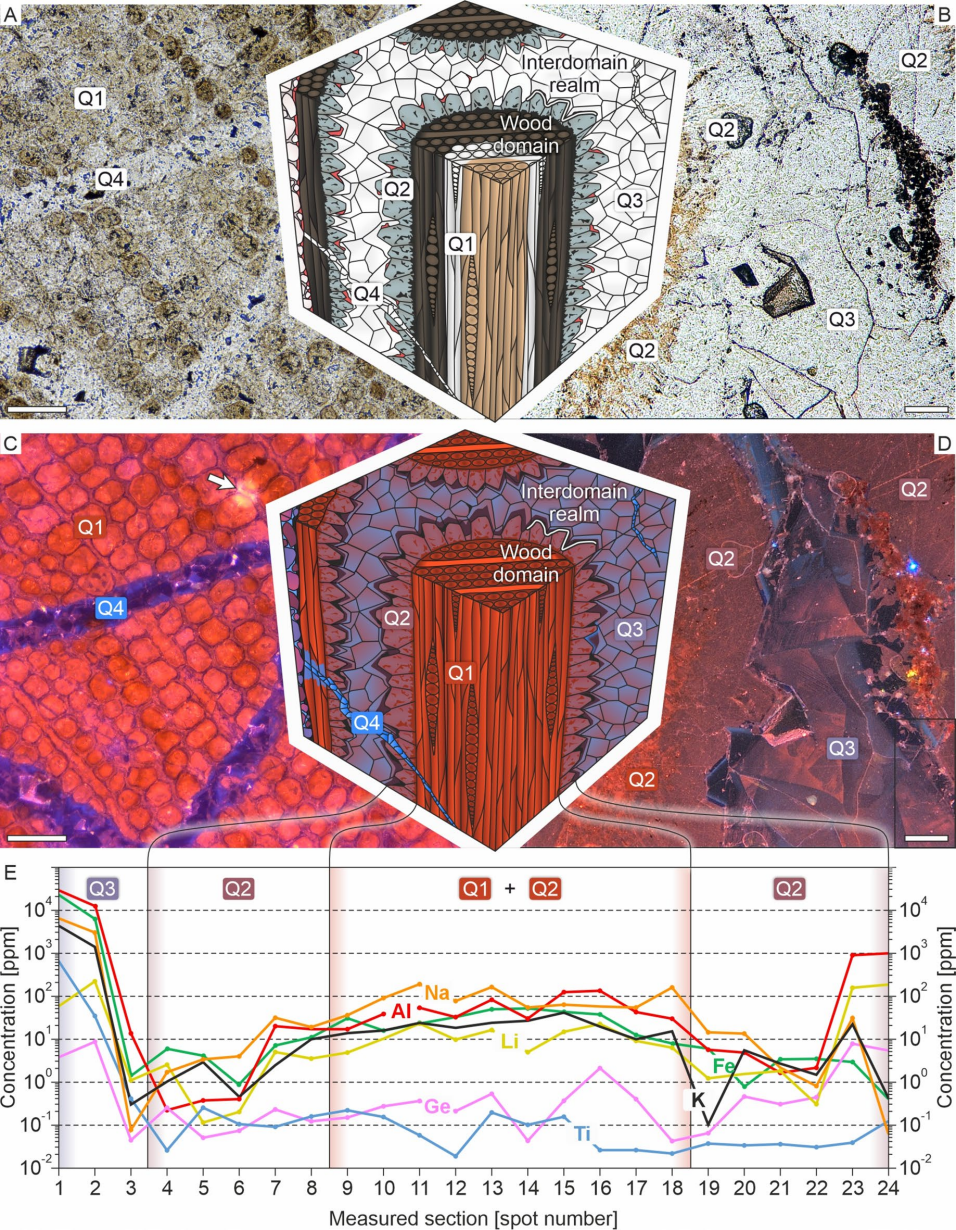
Structural and chemical differentiation of the Kyffhäuser fossil woods. (**A + B**), Transverse thin sections in plane-polarized transmitted light (PPL). (**C + D**), cathodoluminescence photos of (**A + B**). Note the radiation damage halo in (**C**) (white arrow). (**E**), Geochemical profile across a petrifaction domain. Arrows highlight the light blue margin of quartz crystals. (**A + C**): Kelbra 23-11a; (**B + D**): K22-1. Scales: 100 µm. ppm – parts per million.

Compaction is widespread among the fossil stems but reaches from weakly compacted specimens to logs in which the vertical diameter has been reduced by 70% of the original value (Fig. 2A). The trunks typically exhibit a red hematite coating with dark/white mottling on cut surfaces. This mottled appearance – traditionally referred to as ‘pointstone’ or ‘spotstone’^14^ – results from dark, pencil-like domains of permineralized wood that loosely or densely run through the fossil trunks in axial direction (Figs. 2C, 3). While cells are preserved only within these permineralized-wood domains, the space between them is typically filled with blocky, whitish to clear quartz (Fig. 2E) or, rarely, decomposed wood (Fig. 2D), summarized here as the interdomain realm. If the fossil woods are cut in transverse direction, they consequently appear spotted due to the dark subcircular cross-cuts of the wood domains (Fig. 2C)—an appearance that was frequently mistaken as the loose arrangement of aerial roots found in the stems of contemporaneous tree ferns^25, 26, 27, 28, 29^.

Our study reveals that this ‘pointstone preservation’ is much more differentiated in terms of the structure, geochemistry and age of the silica phases and the preservation of cellular details than previously suspected (Fig. 3). In the silicified trunks from the Kyffhäuser, we recognize five silica generations: the initial permineralization (P) of the Kyffhäuser wood, followed by successive quartz stages (Q1–Q4) with discrete paragenetic sequences of authigenic mineralization.

### Initial permineralization – P (304.3 Ma)

**Fig. 4:**
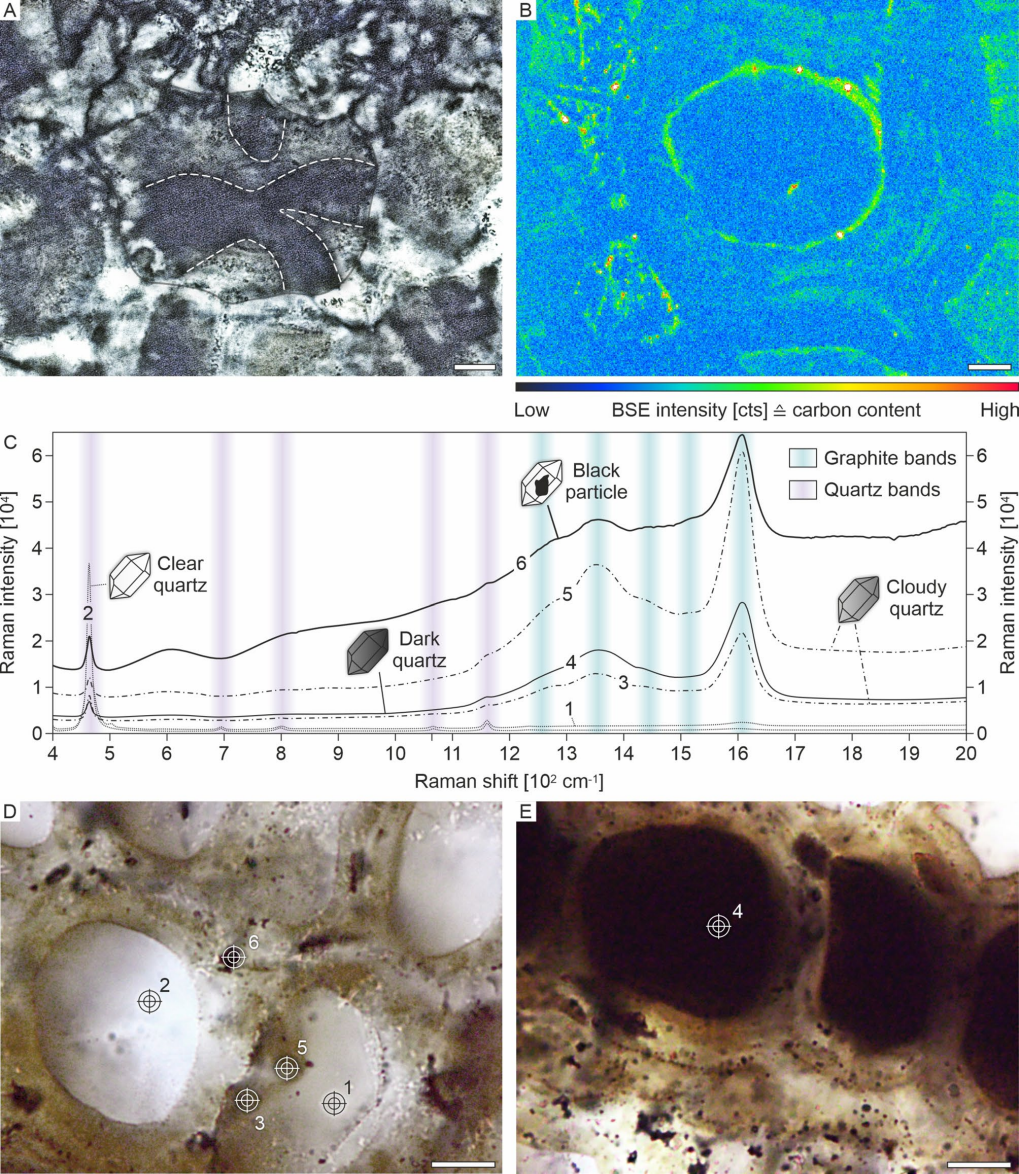
Preservation of organic matter. (**A + B**), Occurrence of graphite in the transverse-cut fossil wood, photo in cross-polarized transmitted light (**A**) and back-scattered electron image (**B**). (**C**), Raman spectra of clear to dark quartz containing various concentrations of graphite. Data shown vertically exaggerated: 1 + 2 (5 ×); 6 (2 ×). (**D, E**), Raman spots in the silicified wood; images in plane-polarized transmitted light. All images from K22-1. Scales: 10 µm (**A**, **B**); 20 µm (**D**, **E**).

Scanning-electron microscope (SEM) imaging and photomicrographs of well-preserved wood cells reveal ultrastructures that are typical for amorphous silica deposits in the cells. These structures comprise multiple silica layers templating the cell walls and lepispheres in the cell lumens (Figs. 4A, B). However, Raman spectra indicate that the originally amorphous phases have been transformed into fine-crystalline quartz (Fig. 4C), thereby implying that the ultrastructures must be regarded as relic evidence of initial silicification. This first mineralization occurred as permineralization, based on relic organic matter within the multi-layered cell walls detected by energy-dispersive X-ray spectroscopy (EDS) and electron-probe micro-analysis (EPMA) (Fig. 4B). The organogenic remains are nowadays preserved as graphite, shown by typical Raman spectra (Fig. 4C–E; Supplementary Data 2), due to thermal maturation during subsequent burial diagenesis^23^.

LA-ICP-MS spot analyses of permineralized wood from the lower Kyffhäuser section yielded a weighted average ^238^U/^206^Pb age of 304.3 ± 9.5/10.5 Ma (Supplementary Data 3). The close match of stratigraphic age and permineralization age attests to silica precipitation shortly after deposition and prior to advanced decay of dead wood.

### Eogenetic opal-quartz transformation – Q1 (299–290 Ma)

Apart from relic ultrastructures related to initial permineralization, the Kyffhäuser wood typically consists of fine-crystalline quartz that fills cell lumens and builds up cell walls. This quartz is termed Q1 here and has intense orange–reddish cathodoluminescence (CL) (Fig. 3C), for which an emission wavelength of around 600 nm was measured^4^. Additional typical spectroscopic features are large, circular spots of up to 50 µm with bright orange luminescence (Fig. 3C), representing radiation-damage halos caused by uranium-bearing mineral inclusions^30^. Q1 shows concentrations in Na, Al, Fe, K, and Li, mostly in the 10–100 ppm range (Fig. 3E; Supplementary Data 4), while Ti and Ge concentrations, by contrast, remain below 2 ppm or even below the detection limit. Primary fluid inclusions of Q1 are monophase at room temperature, corresponding to a trapping temperature below 70 °C, in agreement with the low Ti content (Fig. 3E).

**Fig. 5:**
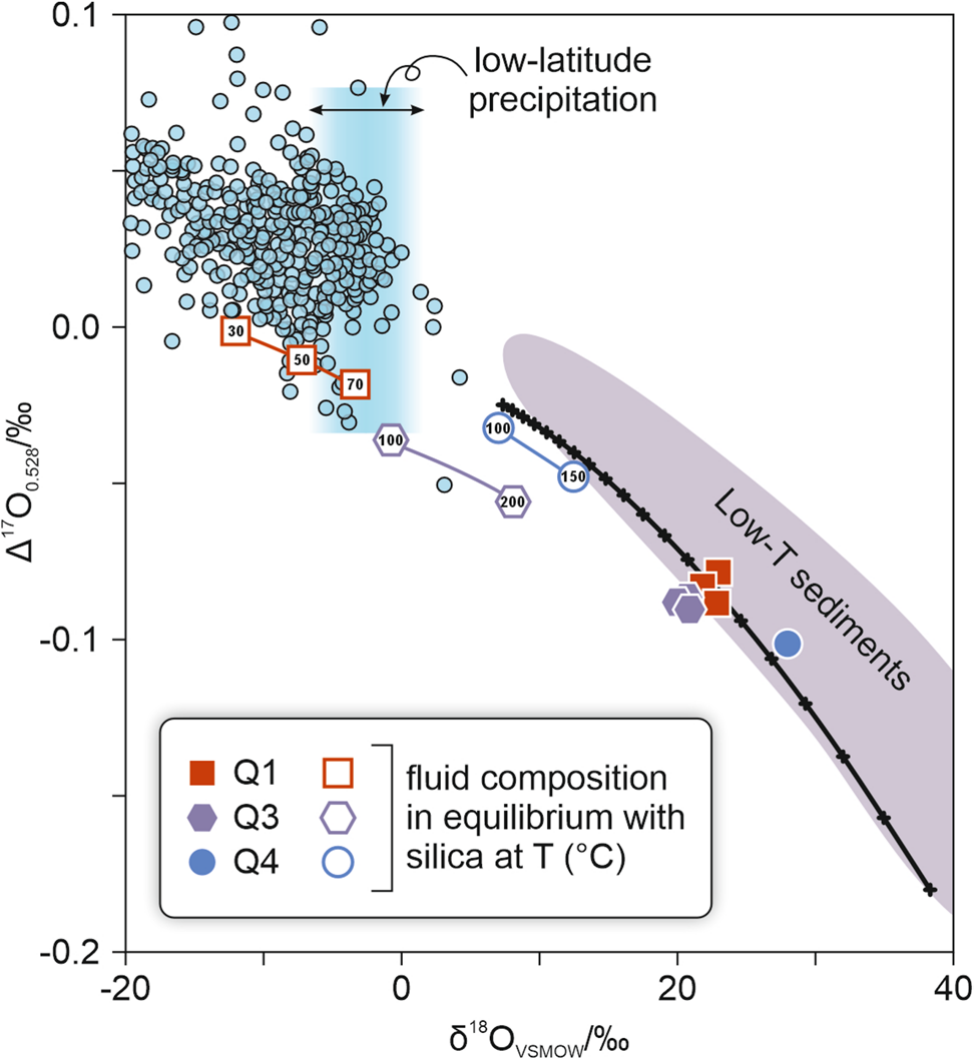
Triple-O isotopes in quartz from silicified wood of the Kyffhäuser, Germany. Solid symbols show the composition of quartz and open symbols the corresponding fluid composition at equilibrium for the temperatures indicated. The low-temperature (T) sediment range is taken from Sharp et al.^34^. Blue data points summarize meteoric fluids^32^.

Silicon isotopes in Q1 are strongly fractionated with δ^30^Si = −2.34, −2.92 and −3.49‰ (Supplementary Data 5), indicating that these signatures are inherited from silica precipitation in a low-temperature environment, where kinetics result in ^28^Si-rich solids^31^. The triple-O isotope composition also plots in the field of sediments formed at low temperature. Assuming formation temperatures between 30°C and 70°C, as documented by the primary fluid inclusions, their equilibrium fluid composition plots in the field of meteoric waters^32^ (Fig. 5). For formation temperatures as high as 50°C–70°C, the reconstructed δ^18^O values fall within the typical range of low-latitudinal precipitation^33^, supporting the view that fluids at depth of Q1 formation still bear a meteoric signature.

**Fig. 6:**
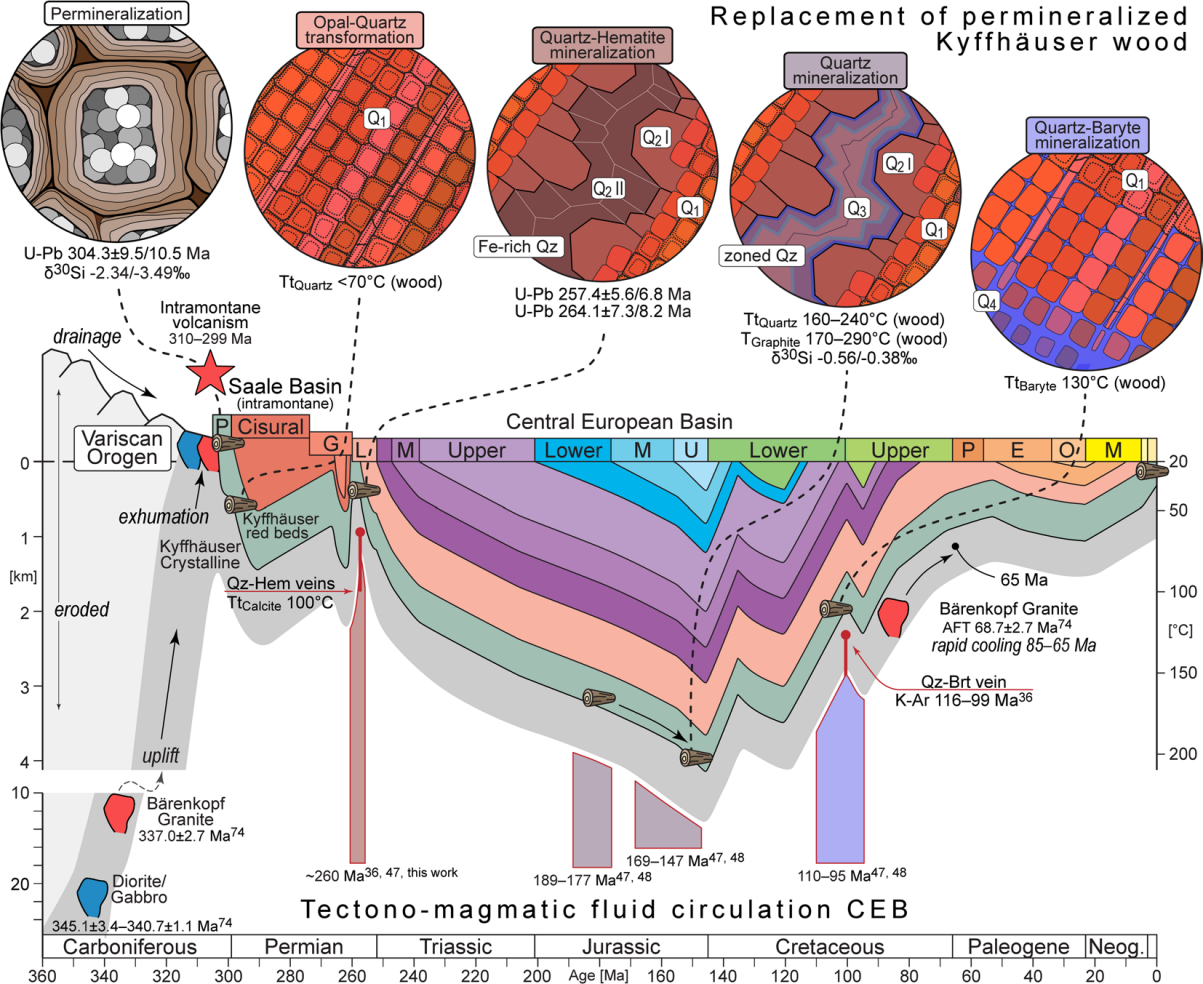
Subsidence history and silicification of the Kyffhäuser fossil wood. Subsidence curve-based late Paleozoic–Cenozoic basin evolution of the Saale Basin, central Germany^23^, modified by evidence for subsidence/uplift recorded in the Kyffhäuser^15, 37^, this work (Fig. 1) and tectono-magmatic phases in the Central European Basin^30, 33, 35, 36, 37, 38^. Abbreviations: AFT – apatite-fission track analysis; Brt – baryte; Cal – calcite; Hem – hematite; Qz – quartz; Tt – trapping temperature.

Because the fine-crystalline quartz of Q1 encloses relics of multi-layered cell walls and lepisphere structures (Figs. 4A, B), the transformation P → Q1 seems to be related to the crystallization of opal to quartz, similar to other examples of silicified wood^12, 35^. Considering anatomical cell preservation (Fig. 4) and the low trapping temperatures of fluid inclusions, the opal-quartz transformation is characterized as an eogenetic, non-destructive phase transition that occurred in shallow burial depth at c. 290 Ma (Fig. 6). The opal-quartz transformation contributed to pervasive silicification of trunks (Figs. 4A, C), an essential taphonomic process that limited mechanical compaction and contributed to anatomical preservation of wood tissues until today.

### Quartz-hematite mineralization – Q2 (257–260 Ma)

The transformation P → Q1 was followed by the replacive paragenetic sequence of quartz and hematite (Q2) coexisting in the Kyffhäuser wood and sandstone, and corresponds to quartz-hematite vein mineralization from basement brines in the Harz and Kyffhäuser^36, 37, 38^. Accordingly, primary two-phase fluid inclusions in calcite from a vein mineralization in the Kyffhäuser Crystalline Complex are highly saline with 75–78% H_2_O, 3–7% NaCl, 17–21% CaCl_2_. Isochore calculations indicate that the inclusions were trapped at c. 100 °C and c. 1 km depth (Supplementary Data 6).

Ascending basement brines stimulated the first replacement of permineralized wood, resulting in the typical pointstone preservation of pencil-like wood domains (Figs. 2E, 3A). Within the pencil-like wood domains, the selective dissolution of fine-crystalline quartz (Q1) and precipitation of up to 500 µm large poikilotopic patches of coarse-crystalline quartz (Q2) contributed to the obliteration of anatomically preserved wood (Figs. 3B, D). Identical radiation-damage halos, luminescence color and element concentrations (Figs. 3C, D) of Q1 and Q2 point to isochemical replacement. LA-ICP-MS spot analyses of coarse-crystalline quartz patches yielded weighted average ^206^Pb/^238^U ages ranging from 264.1 ± 7.3/8.2 to 257.4 ± 5.6/6.8 Ma (Supplementary Data 3), corresponding to vein mineralization ages in the Harz Mountains^39, 40^. This range can be constrained to 257–260 Ma (Fig. 6), as quartz-hematite mineralizations in the Kyffhäuser are present in the basement and Pennsylvanian red beds, but not in the overlying Permian sediments^37^.

The interdomain realm is partially to entirely filled with up to a few millimeters large, sub- to euhedral quartz blades (Q2), forming syntaxial overgrowth on wood domains (Figs. 2E, 3D). The irregular edges of ripped wood tissue preserved within this overgrowth (Fig. 3B) indicate that intense dissolution of permineralized wood (P → Q1) occurred prior to precipitation of euhedral quartz (Q2). Quartz overgrowth on wood tissue exhibits early-stage clear quartz, showing dull orange to dull blue luminescence, followed by late-stage quartz with finely dispersed hematite, showing weak reddish luminescence (Fig. 3D). Element concentrations of clear euhedral quartz are depleted, i.e., Na, Al, Fe, K and Li are mostly below 10 ppm (Fig. 3E).

In parallel, the Kyffhäuser red beds show relics of quartz overgrowth, partly with finely dispersed hematite and dull orange to red luminescence, similar to that of euhedral quartz (Q2) in the fossil wood. The structural relation of quartz grains and overgrowth implies pervasive cementation that was partially or completely replaced by subsequent paragenetic sequences (Fig. 2B).

### Maximum-burial quartz mineralization – Q3 (c. 180–150 Ma)

**Fig. 7:**
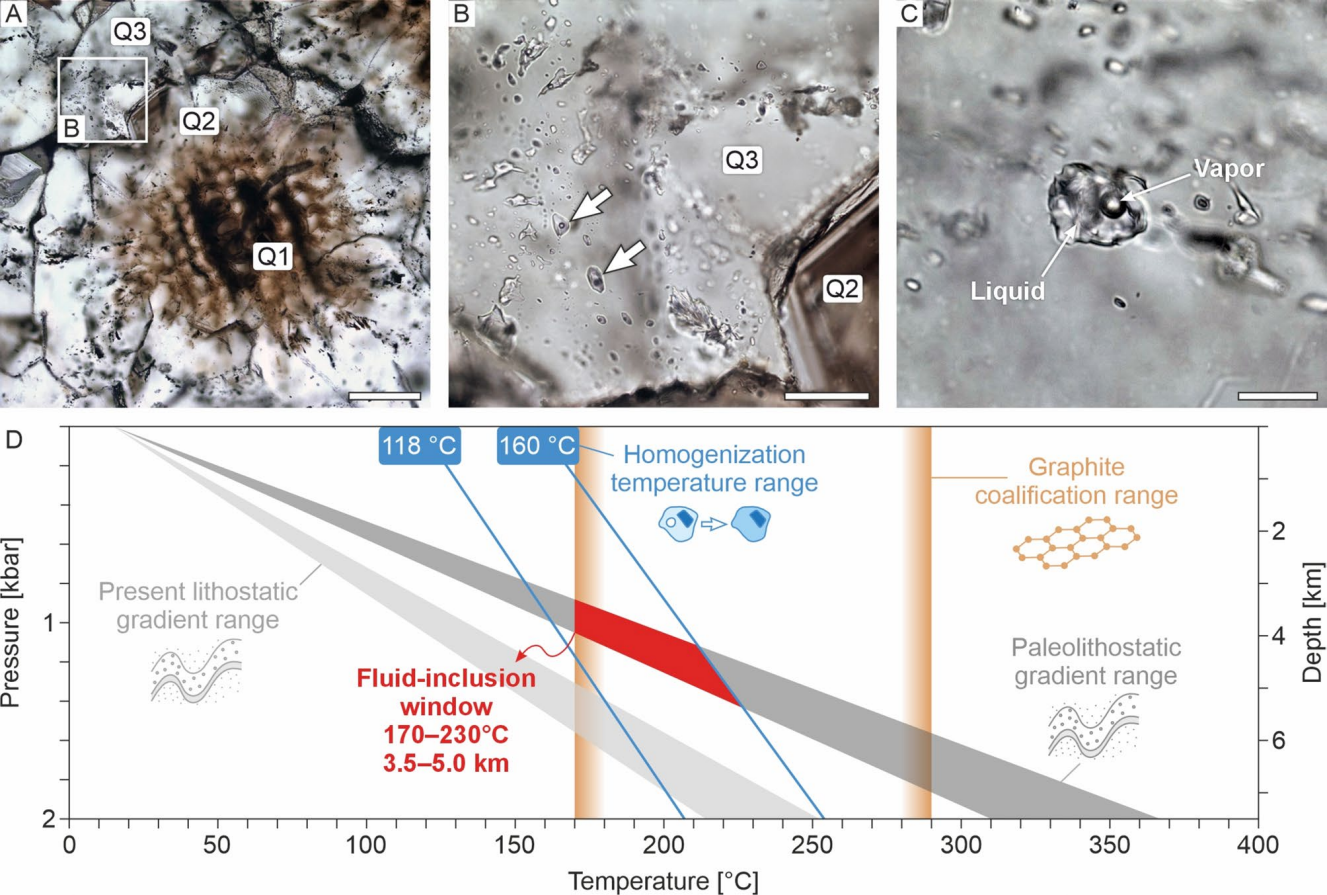
Maximum-burial quartz formation in the Kyffhäuser fossil wood derived from fluid-inclusion analysis. (**A–C**), Occurrence of fluid inclusions in quartz from Q3. (**D**), Multi-parameter calculation of the temperature–pressure conditions during Q3 formation. (**A**–**C**): K22-2. Scales: 200 µm (**A**); 50 µm (**B**); 20 µm (**C**).

The quartz-hematite paragenetic sequence (Q2) was partially to entirely replaced by a generation of up to a few millimeters large, blocky euhedral quartz crystals (Q3) occurring as pore-filling in the Kyffhäuser wood and red beds (Figs. 2B, 3D). The pervasive quartz mineralization of the interdomain realm and intergranular pores is recognized by zoned bluish-purple to reddish luminescence. In the Kyffhäuser silicified wood, element concentrations are elevated, whereby Fe, Al, Na, K and Li contents partly exceed 10,000 ppm (Fig. 3E), indicating alkali-compensated [AlO_4_/M^+^] centers to account for the bluish luminescence^41^. Primary two-phase fluid inclusions in quartz are highly saline basement brines with 71–82 wt% H_2_O, 12–25 wt% CaCl_2_ and 3–12 wt% NaCl; individual inclusions contain muscovite daughter crystals (Fig. 7). Homogenization temperatures of 77–267 °C, with frequency peaks at 120–130 °C and 150–160 °C, point to trapping at 160–240 °C and 0.9–1.2 kbar, corresponding to 3.0–5.5 km depth (Fig. 7).

Raman spectroscopy indicates that organic material transformed to graphite based on the distinctive D and G emission bands between 1,200 and 1,700 cm^−1^ (Fig. 4C). The detected emission bands reflect the presence of various disorder structures in the graphite crystals^42, 43, 44^ and yield peak coalification temperatures between 170 and 290 °C, with a frequency maximum at c. 270 °C.

Si isotopes in Q2 + Q3 corroborate formation at high temperature. Quartz is minimally fractionated (δ^30^Si = 0.56‰ and 0.38‰) relative to the composition of the continental crust (0.22‰^45^), consistent with minimal isotopic fractionation for quartz formation at high temperatures^46^. The inferred fluid O-isotope composition for crystallization temperatures between 100 and 200 °C plots outside the field of meteoric waters and likely represents the composition of a basinal fluid with a complex history.

The quartz mineralization (Q3) in the Kyffhäuser wood partly overlaps in age with economically important vein mineralizations in the Harz dated to c. 190–180 Ma^40, 47^ (Fig. 6). These mineralizations are related to an Early–Late Jurassic rift-related tectono-magmatic phases^48, 49^ and fluid-driven authigenesis in the Variscan basement^50^ and the Carboniferous–Permian strata^51, 52^.

### Quartz-baryte mineralization – Q4 (c. 100 Ma)

The last generation of replacive coarse-crystalline quartz (Q4), showing intense blue cathodoluminescence, occurs as pore fillings and grain overgrowth in the Kyffhäuser red beds and as vein mineralization cross-cutting the Kyffhäuser wood fossils (Fig. 3C). The same intense blue luminescence is seen locally along altered margins of wood fossils in contact with the Kyffhäuser red beds. Within these mm-thick whitish alteration rims, dissolution–precipitation processes led to the gradual replacement of permineralized wood, which is delineated by shifting luminescence colors. In the Kyffhäuser wood and sandstone, replacive quartz is associated with massive baryte mineralization, often attached to the fossil wood, and calcite-vein mineralizations and pore fillings. Primary two-phase fluid inclusions trapped in baryte are highly saline, with 71–79 wt% H_2_O, 24–27 wt% CaCl_2_ and 2–3 wt% NaCl, and indicate trapping at 130 °C and 2.2 km. The quartz-baryte mineralization (Q4) in the Kyffhäuser wood corresponds to the baryte-fluorite vein mineralization of the Harz and Kyffhäuser basements^24, 37^. In the latter, hydrothermal alteration, preceding vein mineralization, is dated to an interval ranging from 116 ± 2.9 Ma to 99 ± 2.2 Ma^37^, corresponding to an Early Cretaceous tectono-magmatic phase of intra-basin magmatism^48, 49^ and fluid-driven authigenesis^39, 50, 53^.

The altered margins of the Kyffhäuser woods are reset in both Si- and O-isotope ratios relative to the interior, optically unaltered region. δ^30^Si is at −0.96 ± 0.11 ‰ and thus relatively enriched in ^30^Si (Supplementary Data 5). The inferred fluid O-isotope composition corresponds to crystallization temperatures between 100 and 150 °C (Fig. 5).

## Discussion

### Basin history from mineralized wood

Silica structure and geochemistry in the Kyffhäuser fossil wood document five stages of silica permineralization or replacement spanning 200 Myr from the Late Pennsylvanian to the Early Cretaceous. If Cenozoic exhumation to surface levels is taken into account, this interval even expands to 300 Myr. This is the longest documented sequence of subsequent wood mineralization stages, although only stages P and Q1 preserved cell structures while stages Q2–Q4 contributed to their destruction.

**Fig. 8:**
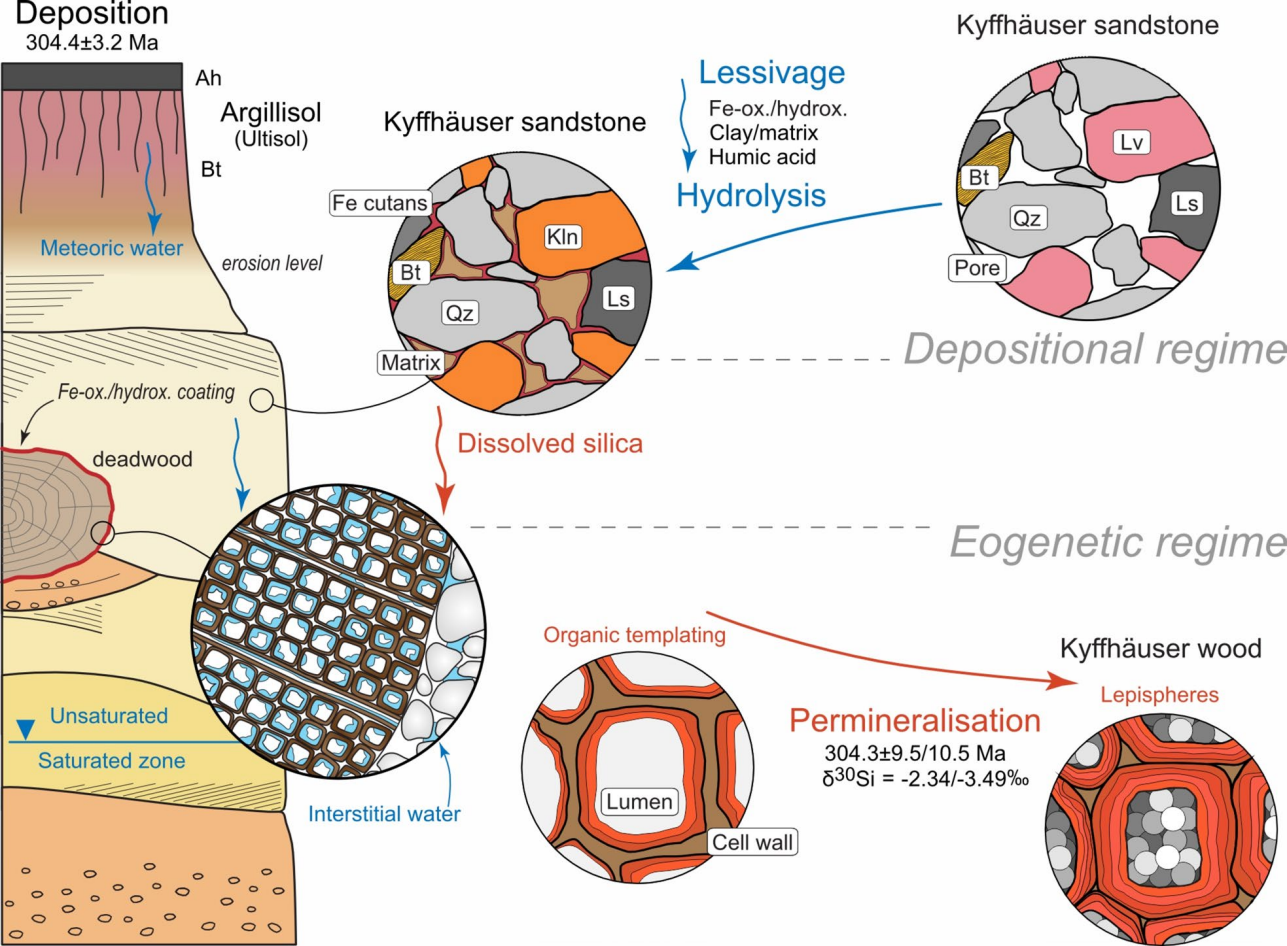
Post-sedimentary permineralization of the Kyffhäuser fossil trunks through pedogenic silica mobilization. Argillisol: Ah – organic-matter soil horizon, Bt – argillic B horizon (illuviation with clay); Minerals: Bt – biotite, Kln – kaolinized lithics, Ls – lithics (general), Lv – volcanic lithics, Qz – quartz.

Genuine preservation of cell structures occurred within the few million years following burial, setting a first taphonomic window for the silicification of woody debris in epiclastic, abrasive environments (Fig. 6). Mineralization of woody debris in sandy bedforms began with early post-sedimentary opalization (stage P) in the Late Pennsylvanian, as relic lepisphere structures, cell-wall linings, and a U–Pb radiogenic isotope age of 304.3 ± 9.5/10.5 Ma document (e.g., Fig. 4A). Given that relic organic carbon in the cell walls was preserved as graphite (Figs. 4C–E), initial silicification in the Kyffhäuser woods occurred as permineralization, i.e., the cell walls were lined rather than replaced (Fig. 8). On the one hand, this outcome is surprising, given the present-day macro-appearance as petrifactions, which now must be regarded as a diagenetic state. On the other hand, assuming initial permineralization rather than petrifaction aligns well with the reconstructed environment and the physicochemical conditions required to preserve organic tissues. Wood taphonomy and Si and O isotope ratios reveal that the first silicification derived from relatively cool waters with a strong meteoric signature in shallow burial depths, allowing for moderate compaction (Fig. 8). This idea agrees with the origin of the Kyffhäuser woods as large woody debris in fluvial red beds that is construed from extensive facies analysis and biostratinomic studies^15^. In riverbed deposits, pH values are around 7, and redox potentials of the pore waters may be low or even negative – both chemical preconditions for preserving cell structures rather than replacing them^12^. With ongoing deposition and the intercalation of floodplain fines, pedogenesis led to the illuviation of iron oxides and silica from altered, reworked pyroclasts into the deeper, wood-bearing coarse clastics, thereby supporting permineralization (Fig. 8). The pyroclasts derive from synsedimentary volcanism, which is also indicated by a crystal-rich tuff^15, 54^ and euhedral zircons in the Kyffhäuser sandstone which gave maximum depositional ages 304.4 ± 3.2 Ma and 302.8 ± 2.2 Ma (Fig. 1).

The conclusion that synsedimentary volcanism indirectly promoted the permineralization of the Kyffhäuser wood has implications for other large-woody-debris occurrences in Europe. As far as the Variscan intramontane basins are considered, such as in France, the Czech Republic and Poland, late- to post-collisional volcanism may have stimulated similar eogenetic silicifications elsewhere^19, 55, 56^. This idea is further supported by comparable structures and textures of fossil woods from these occurrences and the Kyffhäuser^16^. Regarding silicified wood from settings outside the Variscides, such as the intracratonic Donets Basin/Ukraine, there is only questionable evidence for synsedimentary volcanism in the Permian^57, 58^.

Related to ongoing post-collisional extension and subsidence in the early Permian, and the overburden by the volcanic Halle Formation, the amorphous silica phases in the Kyffhäuser woods transformed into micro-crystalline, cell-sized quartz (stage Q1). This was the final phase of cell preservation and ended with compact silicified stems, probably in the early Permian [6, 58]. The following cell-destructive stages Q2–Q4 reflect crucial turning points in the evolution of the CEB. The initial burial was followed by the first inversion (Altmark tectonic phases), as recorded by a widespread hiatus spanning the middle and lower upper Permian across various basins^22^. In the Kyffhäuser, this stratigraphic gap is even larger, as reflected by the upper Permian continental Eisleben Formation and marine Zechstein Group that unconformably rest upon the Pennsylvanian red beds 59; Fig. 1A. Inversion and exhumation of the Kyffhäuser were paralleled by crustal scale fluid flow that stimulated the first destructive replacement stage Q2 dated to 260–257 Ma (Fig. 6). Continuous reburial of the Kyffhäuser sandstone since the latest Permian culminated in a Late Jurassic burial maximum that is consistent with the general extensional regime during the basin fill stage of the CEB^23^. Heating and crustal-scale fluid flow, associated to Mesozoic rift-related volcanism, contributed to organic matter transformation and quartz replacement (Q3) in the Kyffhäuser wood. The second inversion, a stepwise uplift through the Cretaceous and Paleogene, was related to the change from extensional to contractional tectonic regimes that resulted in wide-spread exhumation in central Europe^23, 60, 61^. The initial uplift is reflected by quartz-baryte vein mineralization in the Kyffhäuser basement, dated to 116–99 Ma^37^, and by hydrothermal alteration and quartz replacement (Q4) in the Kyffhäuser wood (Fig. 6). The hydrothermal fluid flow in the Kyffhäuser seems to be associated to a distinct phase of rift-related intraplate volcanism dated to 110–95 Ma in Scania (southern Sweden), the Netherlands and the North Sea^48, 49^. In the Cenozoic, shallow reburial is indicated by marine–terrestrial Eocene to Miocene strata in the northern foreland of the Kyffhäuser and in the Harz Mountains^62, 63^. This was followed by the third inversion and final exhumation in the Miocene–Pliocene that formed the present-day landscape of the Kyffhäuser and Harz Mountains.

The Kyffhäuser fossil trunks demonstrate that fossilized wood can contribute to clarifying local to regional basin development. However, not all silicified plant remains appear to be suitable for this purpose. While the Kyffhäuser wood silica is polyphase and consists of several SiO_2_ generations covering the eogenetic–telogenetic postdepositional stages, there are also monophase silicifications that only record the syndepositional to early eogenetic stages. One example is found at Manebach, about 80 km south of the Kyffhäuser, where early Permian permineralized conifer stems occur within lacustrine stromatolites^64, 65^. The delicate preservation of cell structures is much better than in the Kyffhäuser, and even intracellular fungi can be found. However, attempts to analyze fluid inclusions and to date the Manebach fossils using the U–Pb isotope system did not yield satisfactory results. The early Permian Northern Tocantins Petrified Forest, north-central Brazil, provides abundant silicified debris of woody and non-woody trees in fluvial red beds^66^, thus resembling the Kyffhäuser in terms of environment and trunk biostratinomy. However, petrifaction in Tocantins resulted from silica-saturated groundwater, as represented today by monophase silicifications in the trunks and silcretes^5^. These comparisons show that the suitability of fossil wood for basin analysis depends less on the overall tectonic setting and more on the climatic and sedimentological conditions of its burial. As successful fluid-inclusion analyses in the Qitai Silicified Forest (NW China)^13^ and Kyffhäuser woods demonstrate, polyphase silicifications showing ‘pointstone preservation’ seem most promising for basin analysis. To maximize this potential, however, an investigation of thin sections and polished sections is required that combines various analytical methods, as presented here.

## Methods

### Material selection

Five representative fossil wood and sandstone samples form the basis for this study, chosen based on in-situ sampling in outcrops and ten years of research of c. three dozen of fossil logs in outcrops and mounted specimens in the Kyffhäuser region as well as hundreds of silicified specimens from five public and three private collections^4, 15, 16^. Representative wood specimens meet the two criteria: 1. They show the typical selective tissue preservation of the Kyffhäuser fossil woods referred to as ‘pointstone’; and 2. They provide all mineralization types and morphologies that occur in the Kyffhäuser assemblages, including fracture fillings. All wood samples are trunk pieces of silicified pycnoxylic gymnosperm wood, likely of cordaitalean or conifer affinity^15^. Sandstone samples were taken from outcrop in fossil-wood bearing facies associations of the Kyffhäuser section, both in the vicinity of, and distal from, the embedded silicified trunks. The samples are stored in the public collections of the Museum für Naturkunde Chemnitz (F141, K2097, K5001) and the Georg August University Göttingen (Kelbra 23 and K22 specimens).

### Cathodoluminescence microscopy

Cathodoluminescence (CL) studies of silica cements, both in the fossil woods and the host rock were carried out using a hot cathode HC3-LM-Simon-Neuser CL microscope^67^, equipped with a Kappa DX 40C Peltier-cooled camera. The operating conditions were an acceleration voltage of 14 kV and a beam current of < 1 mA, corresponding to a beam current density of 20–40 μA/mm^2^.

### Fluid-inclusion analyses

Doubly polished sections with a thickness of c. 150–200 µm served for fluid-inclusion studies in quartz, calcite and baryte (for results see Supplementary Data 6). Phase transitions in the inclusions were investigated using a Linkam THMS 600 heating-freezing stage, cooled with liquid nitrogen and coupled to a video system. Stage calibration rested upon a set of synthetic fluid-inclusion standards. The fluid inclusions in these three minerals are completely frozen between ca. −49 and −90 °C. Recorded phase transitions during subsequent warming include (i) eutectic temperatures (Te), (ii) melting of hydrohalite (TmHH), (iii) ice melting (TmI), and (iv) the homogenization temperature (Th). Salinities of the brines were calculated from ice melting temperatures using the equations of Bodnar^68^, whereas the total salinity and Ca/NaCl ratios in the brine were estimated from the model of Steele-MacInnis et al.^69^. Raman analyses of selected fluid inclusions were performed using a Horiba-Jobin–Yvon HR-800 Raman spectrometer equipped with a 488 nm (blue) laser and attached to an Olympus BX41 microscope.

### Electron-probe microanalysis and scanning-electron microscopy

The main trace elements were measured with a JEOL JXA-iHP200F field emission gun Electron Probe Microanalyzer with following detection limits: Ti (7 ppm), Al (17 ppm), K (5 ppm) and Fe (13 ppm) (Supplementary Data 4). The study included BSE, CL imaging, and elemental mapping, notably carbon. High-resolution imaging was performed with a SEM JEOL IT 500 Scanning Electron Microscope, mainly to search for opal structures in the petrified wood.

### Raman CM thermometry (graphite crystallinity)

We employed Raman spectrometry to identify the carbonaceous material (CM) in the quartz and to estimate the degree of graphitization, allowing for calculating a peak temperature of formation^42^. The Raman emission lines consist of the genuine, ordered graphite or G band, and several disordered defect or D bands (bands D1–482^70^). The raw measurements data are included in Supplementary data 2.

### Silicon and oxygen isotope analysis

Si isotopes were analyzed by multi-collector inductively coupled mass spectrometry (MC-ICP-MS) at the Georg August University Göttingen. Sample aliquots of a few milligrams were separated from crushed thick sections and digested using alkali fusion. Solutions were chromatographically purified using DOWEX AG 50 X-8 cation-exchange resin and diluted to 1 µg/mL Si in 0.1 M HCl and doped with 1 µg/mL Mg. Samples and standards were introduced into the Ar plasma via an Apex 2Q desolvator at 71 µL/min using a PFA Nebulizer. Mass bias was corrected for by alternating measurements of the international reference material NBS 28 (sample-standard bracketing). We report the ^29^Si/^28^Si and ^30^Si/^28^Si isotope ratios in delta notation, i.e. the deviation of isotope ratios from NBS 28 (R_sample_/R_NBS 28_ -1) and express isotope ratios in per mil (δ × 10^3^) (Supplementary Data 5). Measurements were conducted in medium-resolution mode (Δm/m = 7000). The long-term reproducibility is 0.11 ‰ (standard deviation: 2σ). The external standards (BHVO-2, Diatomite, JCh-1) are identical to accepted values within our long-term reproducibility 2s.

Oxygen isotopes were analyzed by means of laser fluorination following the protocol described in Pack et al.^71^. About 2 mg samples were heated with a 50 W CO_2_ laser in an atmosphere of BrF_5_. Liberated O_2_ was purified and measured for δ^17^O and δ^18^O. The Δ^’17^O was defined relative to a reference line with slope 0.528. The uncertainty in δ^18^O is about 0.15‰ and in Δ^’17^O about 0.01‰ (standard deviation: 2σ).

### LA-ICP-MS analyses

U–Pb isotopes and trace elements were analyzed by laser ablation–sector field–inductively coupled plasma–mass spectrometer (LA-SF-ICP-MS), using a 193-nm ArF Excimer laser (Analyte Excite + , Teledyne Photon Machines) coupled to a Thermo-Scientific Element XR instrument at Karlsruhe Institute of Technology (KIT). For detrital-zircon dating, two samples of c. 400 g medium-grained sandstone were processed for zircon extraction, from which the youngest age generation served for age calculation (for sample provenance and results see Supplementary Data 1). For U–Pb dating silicified wood of two thick sections (K22-1, K22-2) was searched for appropriate domains high in uranium, and with high ^238^U/^206^Pb ratios. These two criteria were only fulfilled by four domains of intact wood tissue, representing the earliest stage of preservation (stage I). These four domains were analyzed in detail (between 21 and 38 spot analyses) together with the reference glass NIST SRM 61284, and by applying the instrument conditions and tuning parameters listed in the Supplementary Table 1. Subsequently, all raw data were corrected offline, in particular for outliers, utilizing an in-house MS Excel© spreadsheet program^72, 73^. The results of fossil-wood U–Pb dating and trace-element analyses, as well as images of the dated domains, are available in Supplementary Data^74^.

## Supplementary Information

Supplementary Information 1.

Supplementary Information 2.

Supplementary Information 3.

Supplementary Information 4.

Supplementary Information 5.

Supplementary Information 6.

Supplementary Information 7.

## Data Availability

The datasets generated during and/or analysed during the current study are available from the corresponding author on reasonable request.
