# Supplementary material for: Fossil wood cells recorded 300 million years of Europe’s tectonic history
Source: Sci Rep. 2026 Jul 14;16:22068. doi: 10.1038/s41598-026-60054-3 (PMC13369813; doi:10.1038/s41598-026-60054-3)
Supplement: Supplementary file 7 — Supplementary Information 7. [file 41598_2026_60054_MOESM7_ESM.pdf]

Supplementary Table 1. Operating conditions for the LA-ICP-SF-MS equipment for U-Th-Pb isotope analysis

**Laboratory & Sample Preparation**	
Laboratory name	Institute for Applied Geosciences, KIT (Karlsruhe Institute of Technology), Germany
Sample type/mineral	Silicified wood
Sample preparation	Polished thick sections (ca. 250 µm thickness)
**Laser ablation system**	
Make, Model & type	Teledyne Photon Machines, Analyte Excite+ (Excimer)
Ablation cell	Two-volume ablation cell (HELEX 100, EQC COMP), ANUAustralia)
Laser wavelength	193 nm
Pulse width	< 5 ns
Fluence	5.0 J/cm^{-2}
Repetition rate	20 Hz
Spot size	110 µm
Sampling mode / pattern	single spot
Carrier gas	He(cell) 0.30 l/min, He (cup) 0.20 l/min, 0.92 l/min Ar, N_2 (12 ml)
Background collection	15 seconds
Ablation duration	15 seconds
Pre-ablation	3 pulses
Wash-out delay	20 seconds
Cell carrier gas flow (He)	0.50 l/min (total)
**ICP-MS Instrument**	
Make, Model & type	Thermo-Scientific ELEMENT XR (sector field)
Sample introduction	via conventional tubing
RF power	1220 W
Sampler, skimmer cones	Ni-Jet
Extraction lenses	X type
Make-up gas flow (Ar)	0.92 l/min
Detection system	single collector secondary electron multiplier
Data acquisition protocol	Time-resolved analysis
Scanning mode	Peak hopping, four point per peak
Detector mode	Pulse counting mode
Masses measured	^{202}Hg,^{204}(Hg + Pb), ^{206}Pb, ^{207}Pb, ^{208}Pb, ^{232}Th, ^{238}U
Integration time per peak	10 milliseconds
Sensitivity / Efficiency	31500 (cts/ppm), (^{238}U, NIST612, spot-20µm, 10µm/s, 10 Hz, 5 J/cm^2)<br>oxide formation rate: U/UO <0.08, Th/U = 0.98
**Data Processing**	
Gas blank	15 seconds on-peak
Calibration strategy	NIST SRM 612 glass was used as primary reference material, assuming a similar ablation behavior like the silicified wood tissue (matrix offset = 1).
Reference Material info	NIST SRM 612 glass (Pearce et al., 1997).
Data processing package used	Macro-based in-house EXCEL spreadsheet (Gerdes and Zeh, 2006, 2009), modified by RA.Roper at FIERCE Frankfurt am Main

(a) Operating conditions for the LA-ICP-SF-MS equipment for trace element analysis

**Laboratory & Sample Preparation**	
Laboratory name	Institute for Applied Geosciences, KIT (Karlsruhe Institute of Technology), Germany
Sample type/mineral	Silicified wood
Sample preparation	Polished thick sections (ca. 250 µm thickness)
Imaging	Polarized microscope at KIT
**Laser ablation system**	
Make, Model & type	Teledyne Photon Machines, Analyte Excite+ (Excimer)
Ablation cell	Two-volume ablation cell (HELEX 100, EQC COMP), ANUAustralia)
Laser wavelength	193 nm
Pulse width	< 5 ns
Fluence	7.0 J/cm^{-2}
Repetition rate	10 Hz
Spot size	40 µm
Sampling mode / pattern	single spot
Carrier gas	He(cell) 0.30 l/min, He (cup) 0.20 l/min, 0.89 l/min Ar, N_2 (12 ml)
Background collection	25 seconds
Ablation duration	30 seconds
Pre-ablation	0 pulses
Wash-out delay	25 seconds
Cell carrier gas flow (He)	0.50 l/min (total)
**ICP-MS Instrument**	
Make, Model & type	Thermo-Scientific ELEMENT XR (sector field)
Sample introduction	via conventional tubing
RF power	1240 W
Sampler, skimmer cones	Ni-X-cone
Extraction lenses	X – type
Make-up gas flow (Ar)	0.90 l/min
Detection system	single collector secondary electron multiplier
Data acquisition protocol	Time-resolved analysis
Scanning mode	Peak hopping, four point per peak
Detector mode	Pulse counting mode
Masses measured	^{7}Li, ^{23}Na, ^{27}Al, ^{29}Si, ^{39}K, ^{49}Ti, ^{57}Fe, ^{72}Ge; (SiO_2 = 100 wt.% used as internal standard).
Integration time per peak	2-10 milliseconds
Sensitivity / Efficiency	29000 (cts/ppm), (^{238}U, NIST612, spot-20µm, 10µm/s, 10 Hz, 5 J/cm^2) oxide formation rate: U/UO <0.03, Th/U = 0.98
**Data Processing**	
Gas blank	25 seconds on-peak
Calibration strategy	NIST SRM 612 glass,
Reference Material info	NISTSRM 612 (Pearce et al., 1997)
Data processing package used	GLITTER software (van Achterberg, 2000)

References

Gerdes, A., Zeh, A., 2006. Combined U–Pb and Hf isotope LA-(MC-)ICP-MS analyses of detrital zircons: Comparison with SHRIMP and new constraints for the provenance and age of an Armorican metasediment in Central Germany. Earth Planet. Sci. Lett. 249, 47–61.

Gerdes, A., Zeh, A., 2009. Zircon formation versus zircon alteration – new insights from combined U–Pb and Lu–Hf in-situ LA-ICP-MS analyses, and consequences for the interpretation of Archean zircon from the Central Zone of the Limpopo Belt. Chem. Geol. 261, 230–243.

Pearce, N.J.G., Perkins, W.T., Westgate, J.A., Gorton, M.P., Jackson, S.E., Neal, C.R., Chenery, S.P., 1997. A Compilation of New and Published Major and Trace Element Data for NIST SRM 610 and NIST SRM 612 Glass Reference Materials. Geostandards Newsletter 21, 115–144.

van Achterbergh, E., Ryan, C.G., Griffin, W.L., 2000. GLITTER: On-Line Interactive Data Reduction for the Laser Ablation ICP-MS Microprobe. Macquarie University, pp. 61.
